# Attitudes and knowledge toward mental disorders among healthcare providers in psychiatric departments

**DOI:** 10.1192/j.eurpsy.2024.1405

**Published:** 2024-08-27

**Authors:** F. Askri, M. Lagha, K. Ben Younes, I. Ben Romdhane, H. Wided, R. Labbane

**Affiliations:** PSYCHIATRY C, RAZI HOSPITAL, MANOUBA, Tunisia

## Abstract

**Introduction:**

Stigma towards mental health disorders is an issue standing in the way of healing and integrating the patient into the social life. Stigmatising does not only come from the general population but also from health care providers. Studies found out that lack of knowledge and skills among health care professionals is associated with stigmatization, which affects the attitudes and the patient’s treatment process.

**Objectives:**

The main aims of this study were to evaluate the attitudes and knowledge about mental health among nurses and psychiatry residents working in psychiatric departments, to explore the relationship between knowledge and attitude toward mental health and to find out the possible link with sociodemographic and work characteristics

**Methods:**

A cross-sectional questionnaire was conducted in two departments of psychiatry at RAZI hospital focusing on nurses and psychiatrist trainees. The sociodemographic informations, duration and choice of working in psychiatric field, personal experience with mental illness were collected .

The Mental Health Knowledge Questionnaire (MHKQ) and the mental illness clinicians attitude scale (MICA-4 )were used to evaluate the participants mental health knowledge and attitude towards psychiatry and people with mental disorders .

**Results:**

A total of 30 health care providers finished the questionnaire. Their median (± interquartile range) age was 29 (±9)  years within a range of 25 to 60 years old. Our participants were predominantly female (N = 26; 86.7%). The overall median of MHKQ scale was 10 (±6) with a higher score in psychiatrist trainees than nurses but no significant difference was found (p= 0.066) However there was a significant difference between the two groups regarding the MICA scale (p=0.02) with a negative attitude found in the group of nurses . Participants with no personal experience with mental illness along with those who were obliged to work in psychiatric facilities tend to have higher score on the MICA scale with significant statistically relationships, respectively, p =0.18 and p=0.09 We didn’t find any statistically significant relationship between the total scores of the MICA and MHKQ scales ( rho = -0.206, p = 0.275)

**Conclusions:**

In our study negative attitude toward mental disorders were found in the group of nurses. Education about mental health disorders as well as addressing the importance of mental health outcomes must be included in the first year training of every healthcare provider. New strategies Focusing on improving the knowledge and skills among healthcare professionals are important to make due to their positive effect on the recovery.

**Disclosure of Interest:**

None Declared

